# Stressor-induced ecdysis and thecate cyst formation in the armoured dinoflagellates *Prorocentrum cordatum*

**DOI:** 10.1038/s41598-020-75194-3

**Published:** 2020-10-27

**Authors:** Olga Matantseva, Mariia Berdieva, Vera Kalinina, Ilya Pozdnyakov, Sofia Pechkovskaya, Sergei Skarlato

**Affiliations:** Laboratory of Cytology of Unicellular Organisms, Institute of Cytology of the Russian Academy of Sciences, Saint Petersburg, Russia

**Keywords:** Water microbiology, Cellular microbiology, Microbiology, Ecophysiology

## Abstract

Ecdysis, the process of extensive cell covering rearrangement, represents a remarkable physiological trait of dinoflagellates. It is involved in the regulation of the population and bloom dynamics of these microorganisms, since it is required for the formation of their thin-walled cysts. This study presents laboratory data on ecdysis in *Prorocentrum cordatum*, a harmful dinoflagellate species of high environmental significance. We studied external stressors triggering this process and changes in the cell ultrastructure accompanying it. Our experiments showed that mass ecdysis and formation of cysts in *P. cordatum* could be induced by centrifugation, temperature decrease, changes in salinity, and treatment by 2,6-dichlorobenzonitrile, whereas temperature increase, changes in pH and treatment by tetracycline did not have this effect. Obtained cysts of *P. cordatum* did not contain the pellicular layer and were formed in the end of the first stage of this process, i.e. removal of the plasma membrane and the outer amphiesmal vesicle membrane, whereas its second stage, removal of theca, represented excystment. Based on our findings, we conclude that such cysts can be attributed to thecate cysts and suggest *P. cordatum* as a promising model organism for the investigation of cellular and molecular aspects of ecdysis in dinoflagellates.

## Introduction

Dinoflagellates are eukaryotic microorganisms widely spread in marine and freshwater environments over the globe. Being both important primary producers and consumers, they play a tremendous role in the functioning of aquatic ecosystems and biogeochemical cycling of elements. Moreover, dinoflagellates are well known for the ability of many species to form harmful algal blooms and produce potent toxins that can be accumulated by free-living or cultured clams, fish, and other aquatic organisms thus posing a threat to human health^1–3^. Investigating ecology and physiology of these microalgae is one of the research priorities due to their relevance for the aquatic environment management and for the fundamental understanding of ecosystem functioning.

Dinoflagellates are remarkable due to many traits of their morphology and physiology. One of their fascinating features is a complex cell covering consisting of the plasma membrane underlain by amphiesmal vesicles which may contain significant amount of cellulose forming thecal plates (armoured species) or no/minor amount of cellulose (naked species). In some dinoflagellate species, an additional non-membrane pellicular layer is present inside amphiesmal vesicles^4–8^. Both armoured and naked species are capable of cell covering rearrangement in response to stressful environmental conditions^7^. During such a rearrangement amphiesmal vesicles fuse, a cell discards its plasma membrane, the outer amphiesmal vesicle membrane, thecal plates (if present) and forms a new plasma membrane from the inner amphiesmal vesicle membrane. Subsequently, new amphiesmal vesicles appear. Shedding of membranes and thecal plates (if present) is called ecdysis (see^8^, and references therein). This process was shown to occur in many dinoflagellate species, e.g. in *Heterocapsa niei*^9^, *Kryptoperidinium foliaceum* (syn. *Glenodinium* *foliaceum*)^10^, *Scrippsiella hexapraecingula*^11,12^, *Amphidinium rhynchocephalum*^7^, and *A. carterae*^13^. Dinoflagellate cells produced via ecdysis often represent thin-walled cysts, also called “temporary,” “ecdysal” and “pellicle” by various researchers^14^. According to the available descriptions, a cell covering of such cysts consists of the plasma membrane (the former inner amphiesmal vesicle membrane of a vegetative cell) and a continuous non-membrane pellicular layer. The pellicular layer of a cyst is a result of the thickening of the pellicular layer that was present already in a vegetative cell, or it emerges in a cyst de novo^5,7,9–12^.

Although ecdysis in dinoflagellates was initially described several decades ago^9,15–18^, it remains enigmatic. Neither molecular mechanisms of this process, nor its physiological relevance is fully known^19^. Meanwhile, ecdysis is unique from the standpoint of cell biology, since restructuring of a cell covering that occurs during this process involves massive membrane rearrangement and production of the new plasma membrane. The physiology and fine mechanisms of ecdysis still need to be elucidated, and a good research model is required to fill this gap.

As for physiological relevance, one of the facts that are already recognised is that ecdysis is involved in the production of thin-walled and in some cases resting (thick-walled) cysts of dinoflagellates^14^. As a process mediating the production of dinoflagellate cysts, it likely plays an important role in the population dynamics of these organisms. Dinoflagellate cysts are believed to have various functions ranging from the organism progression through the life cycle to withstanding unfavorable environmental conditions^20^.

Whereas resting cysts are undoubtedly recognised as labile ‘seed banks’ promoting bloom outbreaks and taking part in their termination^20–22^, the significance of thin-walled cysts in this respect was doubted until recently^23^. However, by the present time, it has become clear that thin-walled cysts also can be involved in the regulation of dinoflagellate bloom dynamics^14,20^. The triggers and physiology of encystment and excystment are therefore important for understanding ecology of these microalgae. The external factors triggering the formation of thin-walled cysts in different dinoflagellates include changes in temperature^23–26^ and light regime^27^, nutrient depletion and culture ageing^28,29^, as well as treatment by indoleamines^30^.

In the present work, we explored ecdysis in the cultured dinoflagellates *Prorocentrum cordatum* (Ostenfeld) Dodge (syn. *P. minimum* (Pavillard) Schiller*.*), a cosmopolitan species that is mixotrophic, potentially toxic, invasive and readily produces harmful algal blooms^31^. Armoured dinoflagellates *P. cordatum* were previously shown to ecdyse in response to centrifugation, temperature drop, application of the cellulose synthesis inhibitor 2,6-dichlorobenzonitrile (DCB)^8,25,32,33^ and to form cysts in the extended absence of light^34^. Most of these studies, however, reported the data on ecdysis or cyst formation as side findings of the main investigation and without considering the interrelation of these processes. Moreover, ecdysis as a population response to stressors was not quantitatively described. Here we carried out standardised experiments to explore the range of clues initiating ecdysis in cultured *P. cordatum* and compare the rates of ecdysis and structural changes that occur in cells undergoing this process in response to different triggers. We discuss the role of the cell covering rearrangement in the cyst formation by *P. cordatum*, environmental significance of this process and the potential of *P. cordatum* to be a model organism for studying cellular and molecular mechanisms of ecdysis.

## Results

### Induced ecdysis in *Prorocentrum cordatum*

**Figure 1:**
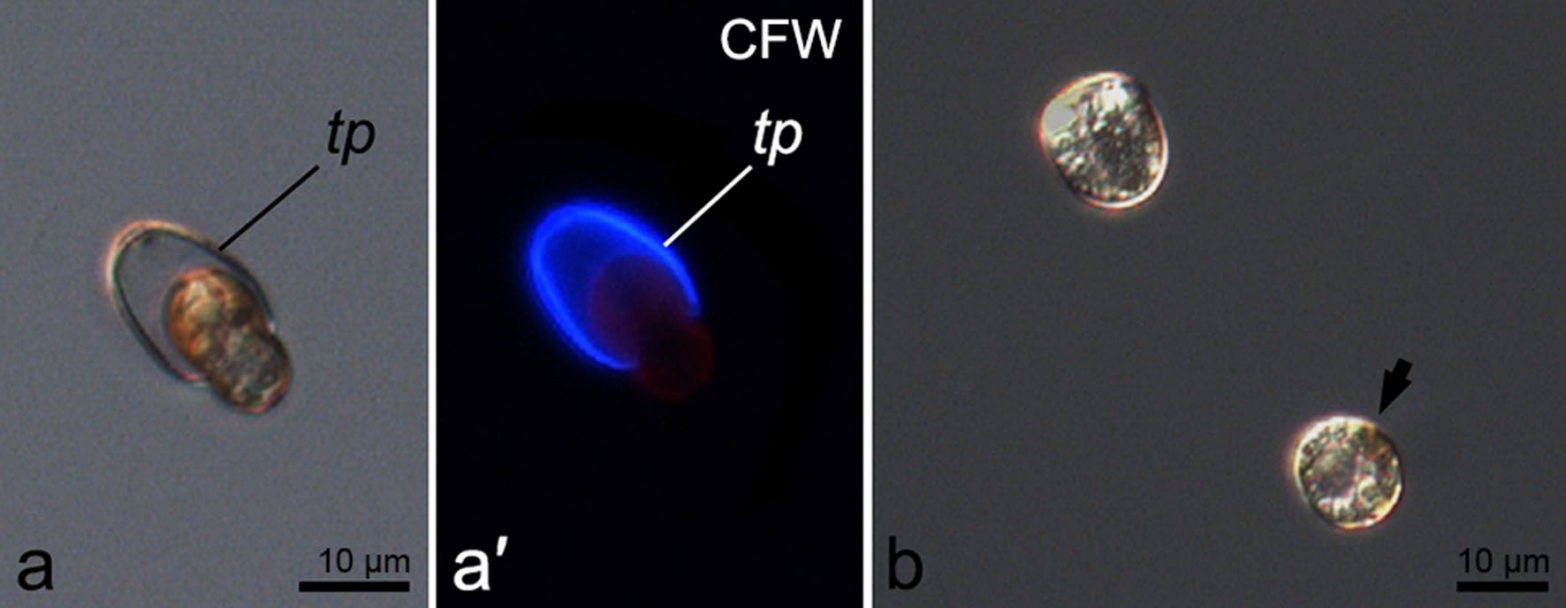
*Prorocentrum cordatum*, RC CCMA 0466 strain, during and after ecdysis. (**a,a′**): cell leaving old thecal plates; (**b**) cell which retained old amphiesma and ecdysed cell (arrow). CFW – Calcofluor White staining, tp – thecal plates.

Ecdysis was induced in the laboratory cultures of *Prorocentrum cordatum* and observed under a microscope. Ecdysing cells lost motility, and their thecal plates could be stained by Calcofluor due to the plasma membrane and outer amphiesmal vesicle membrane damage. After some time (observations in 2–6 h), cytoplasm of such cells slightly shrunk, detached from the valves and then cells escaped their old thecae (Fig. 1a, a′, Supplementary Video 1). Ecdysed cells that had not yet finalised the arrangement of the new full amphiesma looked spherical compared to the cells retaining old thecae and cells with new mature thecal plates (Fig. 1b), but otherwise they appeared to be very similar to vegetative cells and were motile although stressed by heating during the microscopic observation (Supplementary Video 2).

### Various triggers of ecdysis

**Figure 2:**
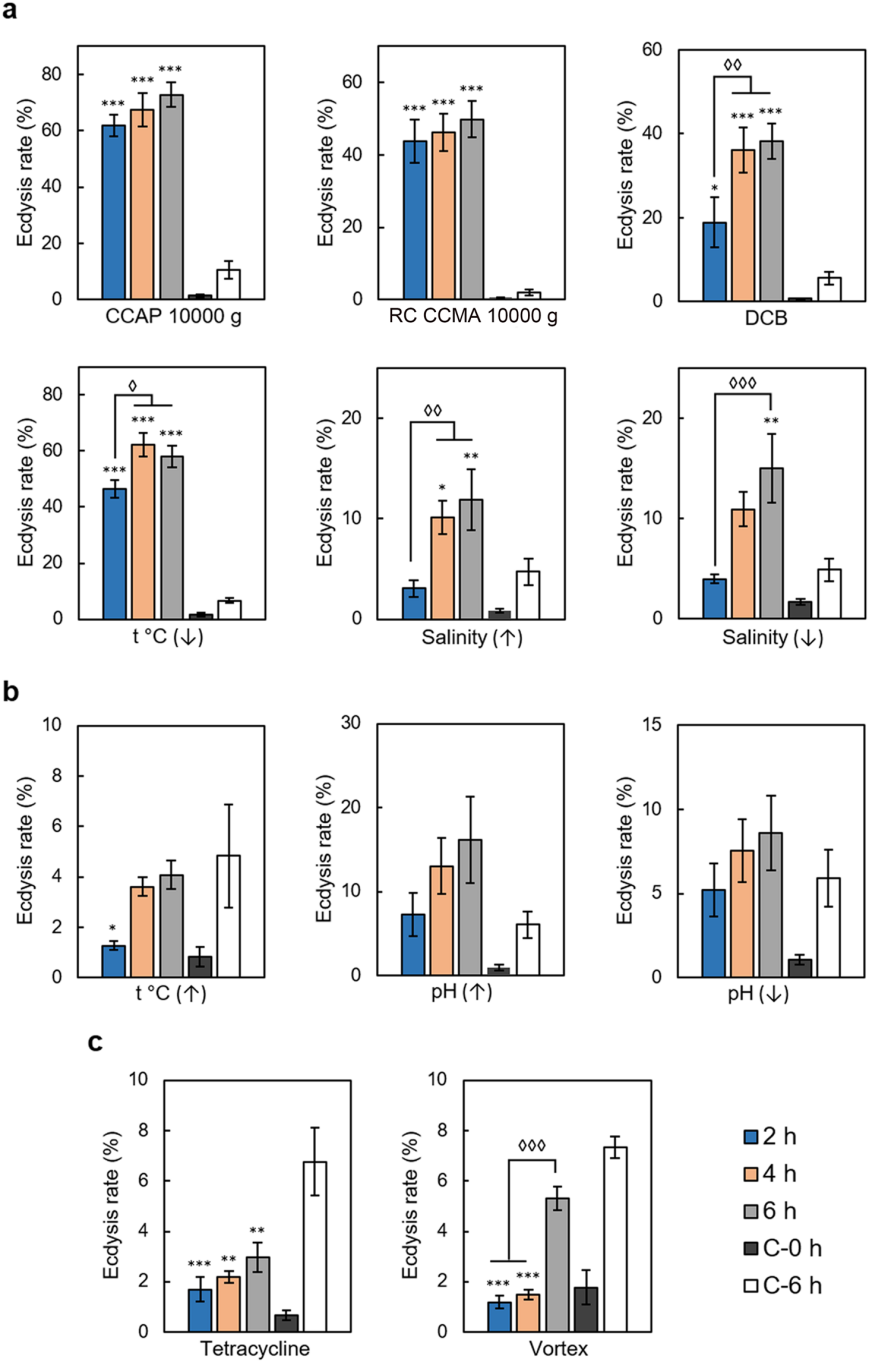
The influence of various factors on the ecdysis rate. (**a**) factors inducing the ecdysis rate; (**b**) factors not affecting the ecdysis rate; (**c**) factors suppressing the ecdysis rate. 2 h – in 2 h after the treatment, 4 h – in 4 h after the treatment, 6 h – in 6 h after the treatment, C-0 h – intact control representing natural ecdysis rate in cultures, C-6 h – procedural control subject to all experimental manipulations excluding the treatment itself (centrifugation). Asterisks indicate statistically significant differences between the control and experimental samples; diamonds indicate statistically significant differences between experimental samples obtained in 2, 4, and 6 h after the treatment (ANOVA with Tukey post-test t, n = 5–7 *, ^◊^*p* < 0.05, **, ^◊◊^*p* < 0.01, ***, ^◊◊◊^*p* < 0.001).

Dinoflagellates at the exponential growth phase with a mean specific growth rate of 0.29 ± 0.05 d^-1^ underwent complete ecdysis including thecae shedding in response to centrifugation at 10,000 g for 5 min, ice cooling for 20 min, treatment with 150 µM DCB, salinity increase to 40 and decrease to 10 (Fig. 2a). The highest rate of complete ecdysis determined by counting of shed thecae (*E*_*thecae*_) was observed in cultures treated by centrifugation (over 60% and 40% for the strains CCAP1136/16 and RC CCMA 0466, correspondingly) and low temperature (over 40%). DCB exposure also resulted in a high ecdysis rate of 20–40% depending on the time point. It must be noted that, similar to the previous findings^29^, treatment with DMSO, a solvent used to dissolve DCB, did not lead to thecae shedding, so the ability to induce ecdysis could be attributed solely to the activity of DCB. In the case of centrifugation, ecdysis rate was not significantly different in samples obtained in 2, 4, and 6 h after the treatment; in the case of DCB treatment it achieved its maximum only in 4 or 6 h after the addition of this chemical. Ecdysis triggered by salinity shifts was detected only in 4 h (salinity decrease) and 6 h (salinity increase) after the treatment and proceeded at a rate of 10–15% (Fig. 2a).

In turn, heating up to 35 ℃ for 20 min, 0.5-step shifts in pH lasting for 20 min, treatment by tetracycline, and vortexing for 1 min did not induce thecae shedding (Fig. 2b, c). Intriguingly, in our experiments treatment by tetracycline and vortexing even lowered the rate of ecdysis (*E*_*thecae*_) as compared to the control, albeit this effect was not statistically significant in 6 h after the treatment in the case of vortexing (Fig. 2c).

**Figure 3:**
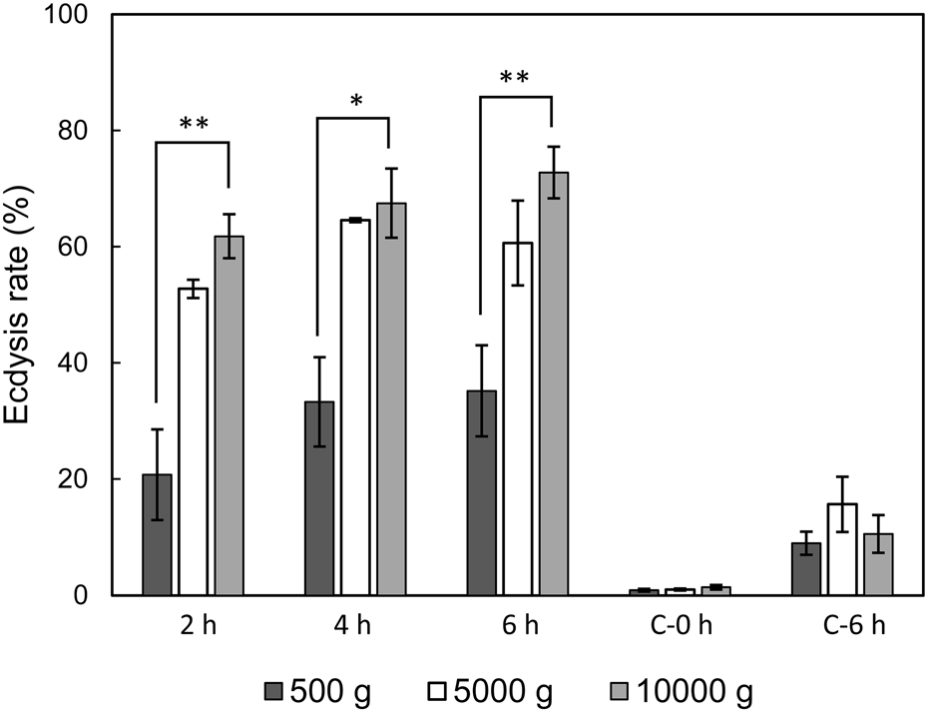
Rate of ecdysis induced by centrifugation of various intensity. 2 h – in 2 h after the treatment, 4 h – in 4 h after the treatment, 6 h – in 6 h after the treatment, C-0 h – intact control representing natural ecdysis rate in cultures, C-6 h – procedural control subject to all experimental manipulations excluding the treatment itself (centrifugation). Asterisks indicate statistically significant differences (ANOVA with Tukey post-test t, n = 3, *p < 0.05, **p < 0.01, ***p < 0.001).

We also tested the effect of stressor strength using the example of centrifugation intensity and found out that centrifugation at 5000 g and 10,000 g equally induced ecdysis, while centrifugation at 500 g resulted in a consistently weaker response (Fig. 3).

### Culture growth phase and ecdysis

Assuming that the growth rate might affect cell susceptibility to a stressor, we determined the rate of ecdysis completion (E_*thecae*_) in response to centrifugation (5000 g, 5 min) in cultures at the different growth phases, i.e. early exponential growth phase with a specific growth rate of 0.24 ± 0.02 d^-1^, late exponential growth phase with a specific growth rate of 0.08 ± 0.03 d^-1^, and stationary growth phase. The experiments did not show any statistically significant differences in the rate of ecdysis completion (*E*_*thecae*_) induced by the same stressor in a culture at different growth phases (ANOVA, p = 0.87).

### Initiation and progression of ecdysis and cell mortality

**Figure 4:**
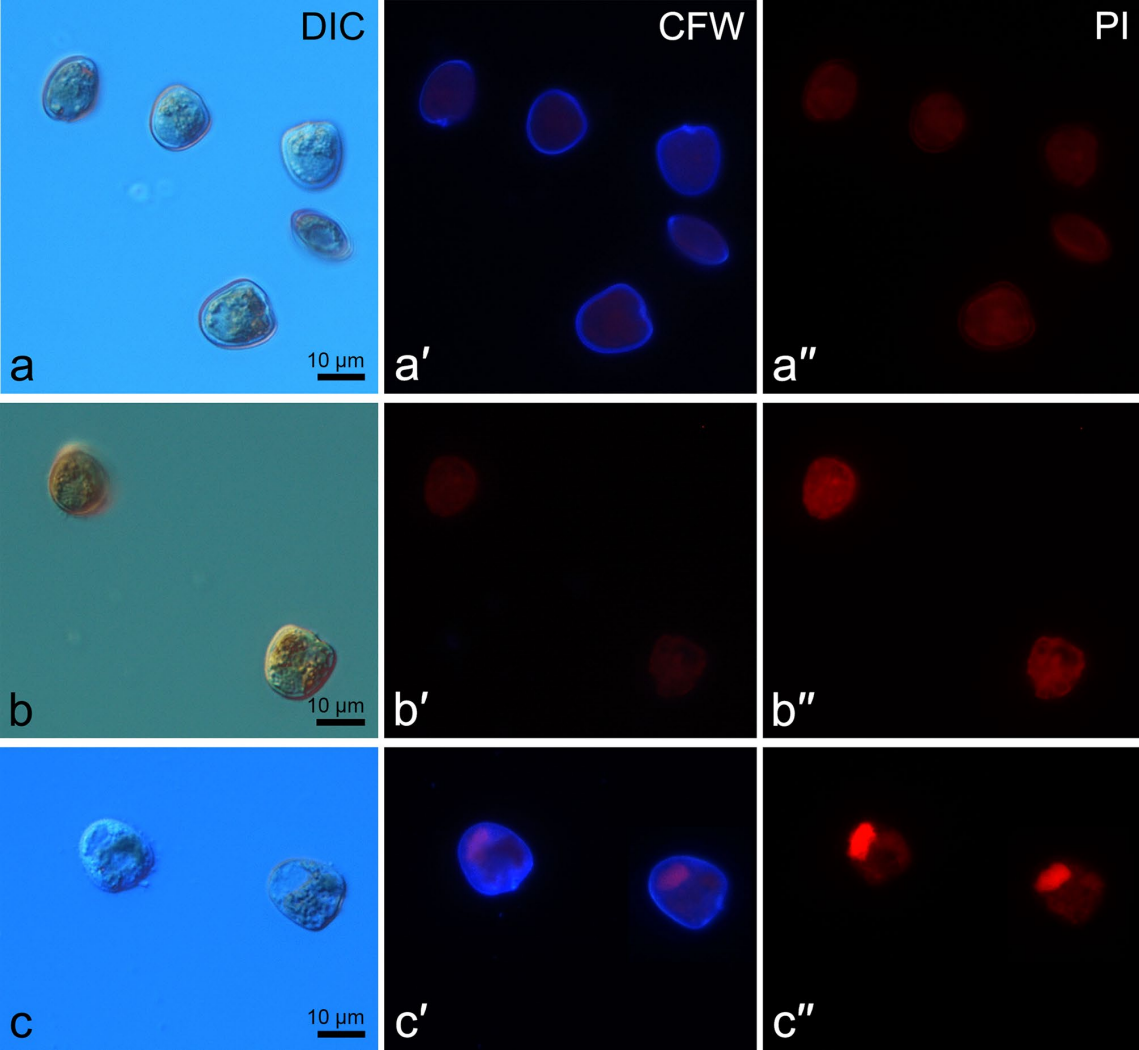
*P. cordatum* cells immediately after the treatments. (**a–a′′**) ecdysing cells after centrifugation (10,000 g, 5 min) visualised by (**a**) differential interference contrast microscopy (DIC), (**a′**) Calcofluor White staining (CWF) and fluorescent microscopy, (**a′′**) propidium iodide staining (PI) and fluorescent microscopy; thecal plates are stained by Calcofluor White due to shedding of the plasma and outer amphiesmal vesicle membranes; (**b–b′′**): immotile cells after vortexing visualised by (**b**) DIC, (**b′**) CWF and fluorescent microscopy, (**b′′**) PI and fluorescent microscopy; no staining is detected; (**c–c′′**): dead cells after lowering the salinity level (from 25 to 10) visualised by (**c**) DIC, (**c′**) CWF and fluorescent microscopy, (**c′′**) PI and fluorescent microscopy; thecal plates are stained by Calcofluor White and nuclei are stained by propidium iodide.

**Figure 5:**
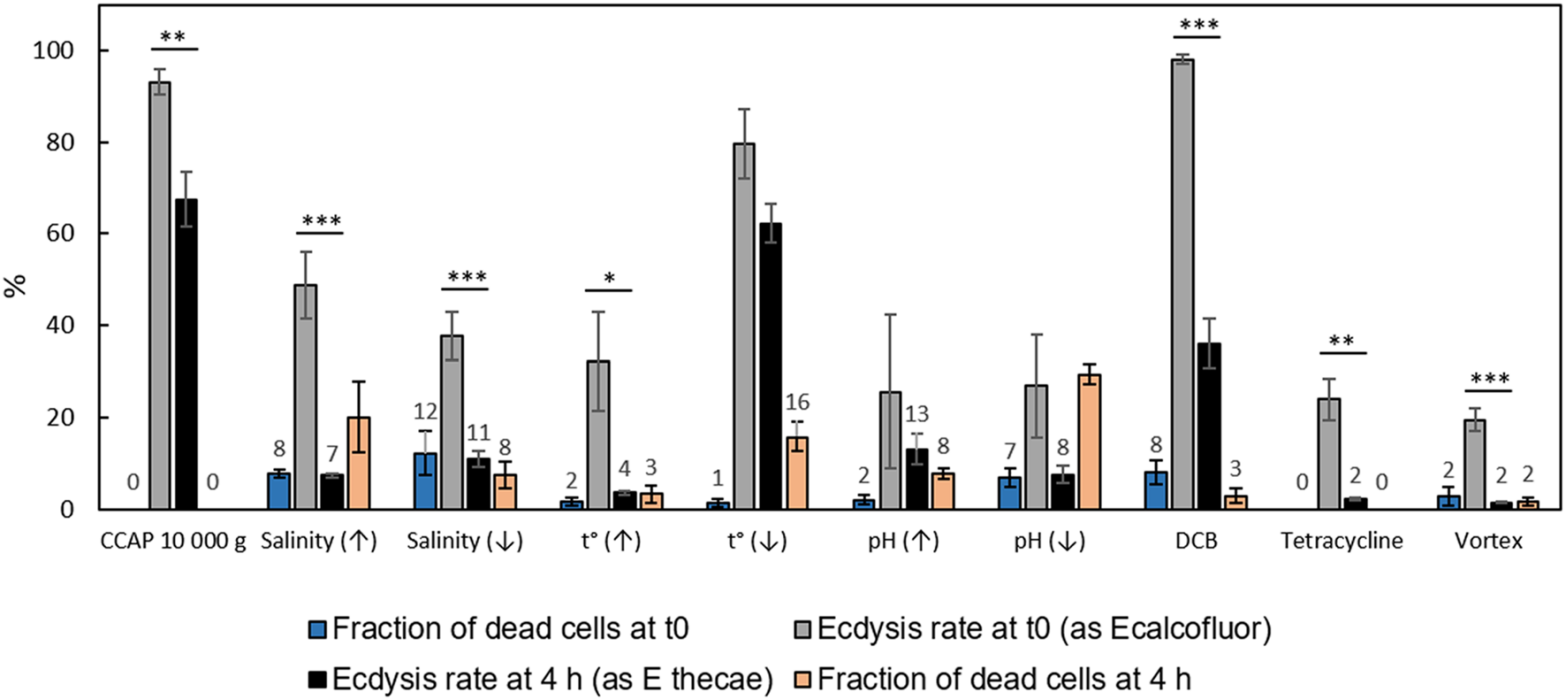
Ecdysis rate immediately and 4 h after the treatments and cell viability. Values below 20 are shown. Asterisks indicate statistically significant differences (unpaired t-test, n = 3, *p < 0.05, **p < 0.01, ***p < 0.001).

Since thecae shedding represents the final stage of ecdysis, *E*_*thecae*_does not characterise the rate of ecdysis initiation associated with shedding of the plasma and outer amphiesmal vesicle membranes. Therefore we estimated the rate of ecdysis initiation immediately after the treatment as the fraction of Calcofluor-stained cells (*E*_*calcofluor*_) and compared it to the rate of complete ecdysis determined in 4 h after the treatment (*E*_*thecae*_) (Figs. 4, 5). Centrifugation at 10,000 g, low temperature and DCB treatment resulted in the highest rate of ecdysis initiation, i.e. over 80% of cells started to discard plasma membranes immediately after the treatment by low temperature and over 90% of cells after the treatments by centrifugation and DCB. At the same time, ecdysis rate (*E*_*thecae*_) at 4 h after the treatment was significantly lower in the case of centrifugation and DCB, indicating that not all cells that had initiated ecdysis shed their thecae and completed ecdysis in 4 h. This trend was typical for most treatments except for ‘low temperature’ and ‘changes in pH’.

In order to make sure that cells survived the applied treatments and subsequent ecdysis we tested cell mortality at 0 and 4 h after the treatment and compared obtained data to the ecdysis rate at the same time points (Figs. 4, 5). Propidium iodide staining demonstrated that the majority of cells were viable in all treatments and at all time points. Immediately after the treatment, the fraction of dead cells stained by propidium iodide did not exceed 12% and was smaller than the fraction of cells that started to ecdyse (*E*_*calcofluor*_). In 4 h after the treatment, the fraction of dead cells increased in samples treated by elevated salinity, low temperature, and pH shifts (Fig. 5). In all other cases, the fraction of dead cells did not change over time. The highest cell mortality was detected in the treatments by salinity changes, low temperature, and pH decrease. In the salinity treatments at the time point ‘4 h’ cell mortality rate was equal or exceeded the rate of ecdysis. In the pH treatment cell mortality rate in 4 h after the treatment exceeded the rate of ecdysis at the same time point. Very low mortality rates were observed in the treatments by centrifugation, high temperature, tetracycline, and vortexing. DCB treatment led to the noticeable mortality rates which were nevertheless much lower than the respective rates of ecdysis at both time points.

A healthy condition of cells was also confirmed by the direct intravital microscope observations and cell counts in 24 h after centrifugation (10,000 g, 5 min). By that time, virtually all observed cells were motile and looking normally, and the cell concentration was the same as immediately after the treatment indicating no cell lysis over time.

### Ultrastructural changes during ecdysis

**Figure 6:**
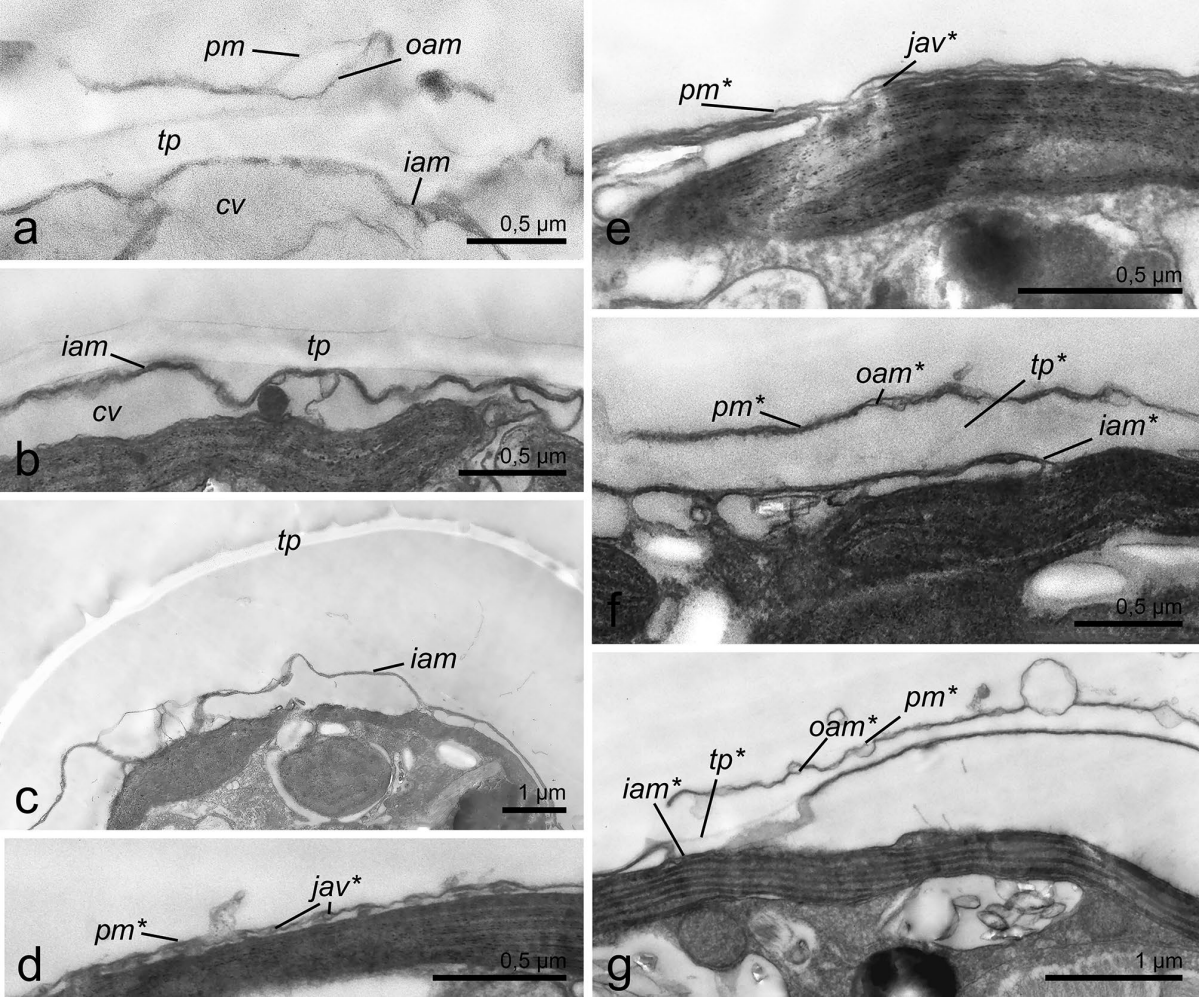
The cell covering rearrangement during and after ecdysis induced by centrifugation (10,000 g, 5 min) in cells of *P. cordatum*, strain CCAP 1136/16. (**a**): native organization of amphiesma; (**b**): shedding of old plasma membrane and outer amphiesmal membrane immediately after centrifugation; (**c**): shedding of old thecal plates; (**d**): accumulation of juvenile amphiesmal vesicles in the cortical cytoplasm; (**e**): formation of new amphiesmal vesicles by fusion of juvenile vesicles; (**f**): formation of new thecal plates; (**g**): maturing of new amphiesma. cv – cortical vesicles, jav – juvenile amphiesmal vesicles, oam, iam – outer and inner amphiesmal vesicle membrane, pm – plasma membrane, tp – thecal plates. New amphiesma elements are marked by asterisks.

**Figure 7:**
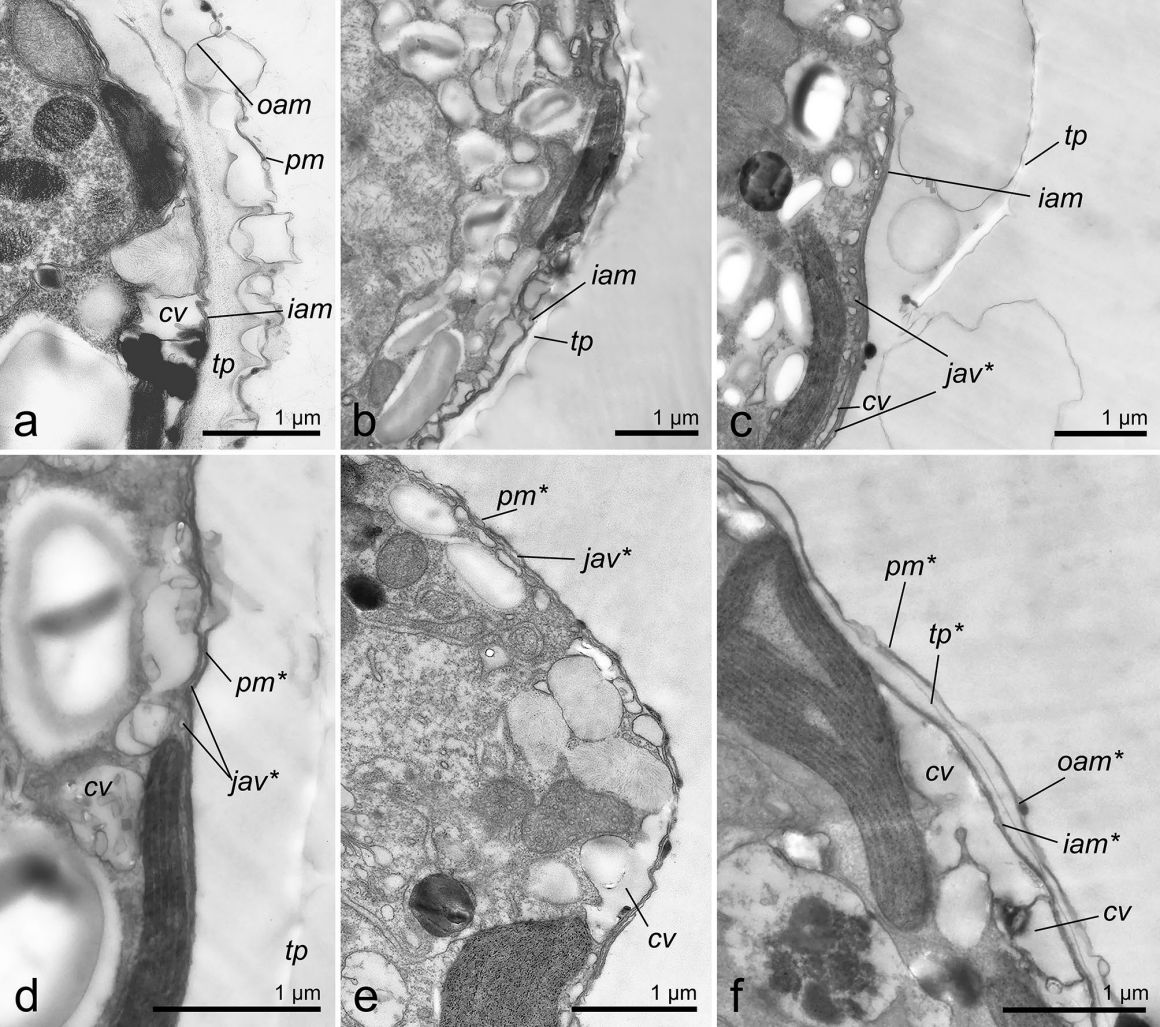
Changes in the fine structure of amphiesma during and after ecdysis induced by centrifugation (10 000 g, 5 min) in cells of *P. cordatum*, strain RC CCMA 0466. (**a**): native organization of amphiesma; (**b**): shedding of old plasma membrane and outer amphiesmal membrane immediately after centrifugation; (**c, d**): shedding of old thecal plates and accumulation of juvenile amphiesmal vesicles in the cortical cytoplasm; numerous small vesicles and tubules are observed in the cortical vesicles; (**e**): formation of new amphiesmal vesicles; (**f**): formation of new thecal plates and maturing of new amphiesma. cv – cortical vesicles, jav – juvenile amphiesmal vesicles, oam, iam – outer and inner amphiesmal vesicle membrane, pm – plasma membrane, tp – thecal plates. New amphiesma elements are marked by asterisks.

**Figure 8:**
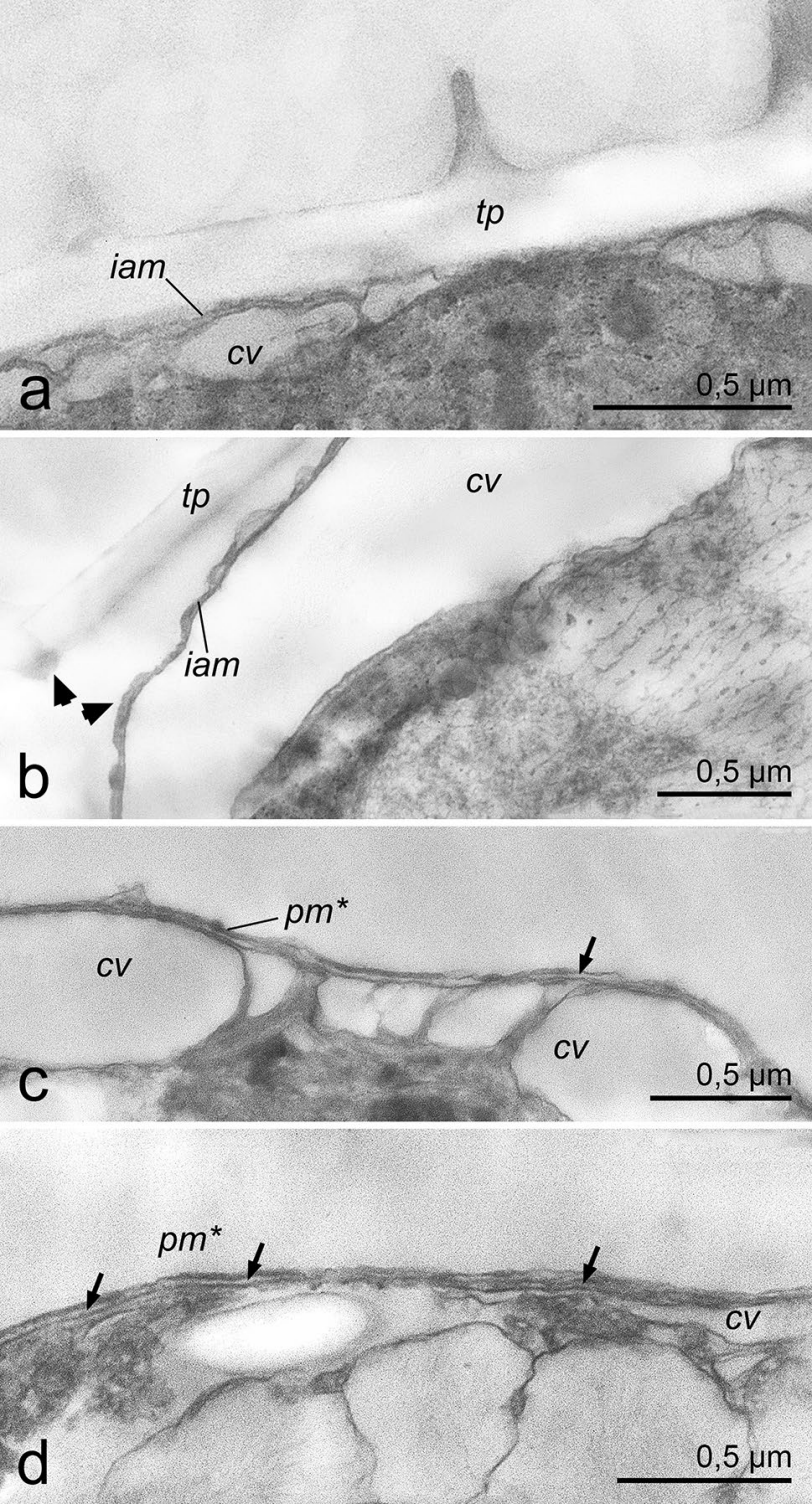
Changes in the fine structure of amphiesma in cells of *P. cordatum*, strain CCAP 1136/16, after DCB treatment. (**a**): cell shedded old plasma membrane and outer amphiesmal membrane immediately after the treatment; (**b**): shedding of old thecal plates, separation point is marked by arrowheads; (**c,d**): 4 h after the treatment, flattened vesicles (arrows) are observed beneath the new plasma membrane. cv – cortical vesicles, iam – inner amphiesmal vesicle membrane, pm* – new plasma membrane, tp – thecal plates.

**Figure 9:**
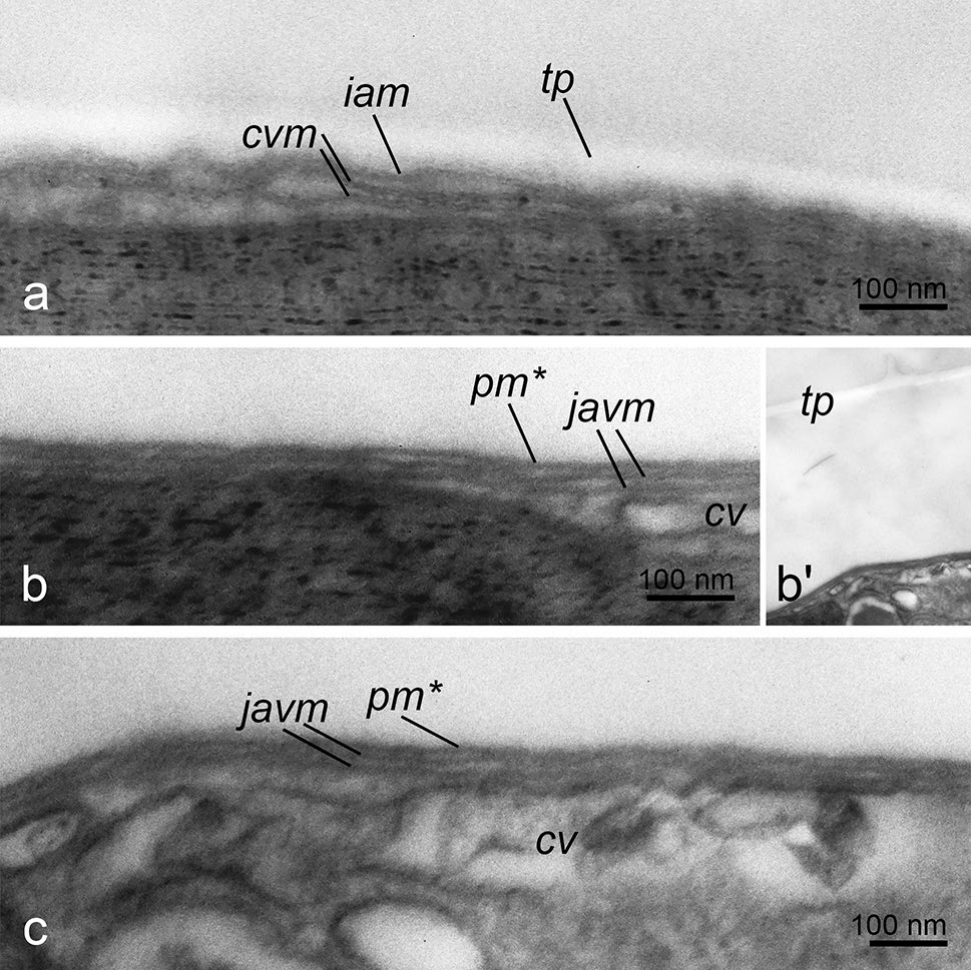
Membrane arrangement in the cells of *P*. *cordatum*, strain CCAP 1136/16, at the different stages of ecdysis induced by centrifugation at 25,000 × magnification. (**a**): cell that shedded the old plasma membrane and outer amphiesmal membrane immediately after centrifugation; (**b,b′**): cell leaving its old thecal plates, a close-up (**b**) and a lower magnification view (**b′**); (**c**): cell that shedded old thecal plates. cv – cortical vesicles, cvm – cortical vesicle membrane, iam – inner amphiesmal vesicle membrane, javm – juvenile amphiesmal vesicle membrane, pm – plasma membrane, tp – thecal plates. New amphiesma elements are marked by asterisks.

TEM images of *P. cordatum* belonging to both studied strains (Figs. 6, 7 for the strains CCAP1136/16 and RC CCMA 0466, correspondingly) demonstrated that before the treatments cells had full prorocentroid amphiesma consisting of the plasma membrane, outer amphiesmal vesicle membrane, cellulose thecal plates and inner amphiesmal vesicle membrane. Moreover, relatively large cortical vesicles could be observed beneath the amphiesma (Figs. 6a, 7a). Immediately after the treatments, i.e. centrifugation and DCB addition, the plasma membrane and outer amphiesmal vesicle membrane were discarded leaving thecal plates exposed to the environment (Figs. 6b, 7b, 8a, 9a). The cells remained covered by thecal plates and the former inner amphiesmal vesicle membrane (the new plasma membrane). No additional layer that could be interpreted as the pellicular layer was revealed either outward or inward from thecal plates and the new plasma membrane. High magnification electron micrographs showed that the membranes which were observed under thecal plates were the former inner amphiesmal vesicle membrane (the new plasma membrane) and closed membranes of cortical vesicles with a thickness of 7–10 nm typical for the cellular phospholipid membranes (Fig. 9a).

Samples incubated for 4 h after the treatment contained cells of various morphology related to the different stages of the cell covering rearrangement. Among them, we observed cells with flaking off thecal plates (Figs. 6c, 7c, d, 8b, 9b–b′). Again, no additional continuous layer above the inner amphiesmal vesicle membrane (the new plasma membrane) was revealed on the micrographs (Figs. 6c, 7c, d, 8b, 9b, c). The only continuous layer with a thickness of 7–10 nm that was observed in the cells leaving their old thecae (Fig. 9b) and cells that have already discarded thecal plates (Fig. 9c) was the new plasma membrane (the former inner amphiesmal vesicle membrane). The membranes distinguishable beneath it belonged to the vesicles of different sizes which we interpret as maturating new amphiesmal vesicles (juvenile vesicles) and cortical vesicles (Fig. 9b, c). Juvenile amphiesmal vesicles were present in the cortical cytoplasm of many ecdysed cells in the 4-h samples (Figd. 6d, 7c, d, 8c). According to the obtained micrographs, these vesicles began to accumulate already in the cells leaving thecal plates (Fig. 7c, d). In the 4-h samples, we observed cells with tiny juvenile amphiesmal vesicles beneath the new plasma membrane (Figs.6d, 7c, d, 8c) and cells with the larger ones (Figs. 6e, 7e, 8d, 9c). We assume that the larger vesicles are a result of the fusion of the smaller vesicles. Finally, in the same samples, we found cells with the almost mature new amphiesma, i.e. amphiesma of such cells included large amphiesmal vesicles containing newly synthesised thin cellulosic thecal plates (Figs. 6f.,g, 7f.). Notably, accumulation of cellulose and the formation of new thecal plates were not observed in cells treated by DCB (Fig. 8c).

**Figure 10:**
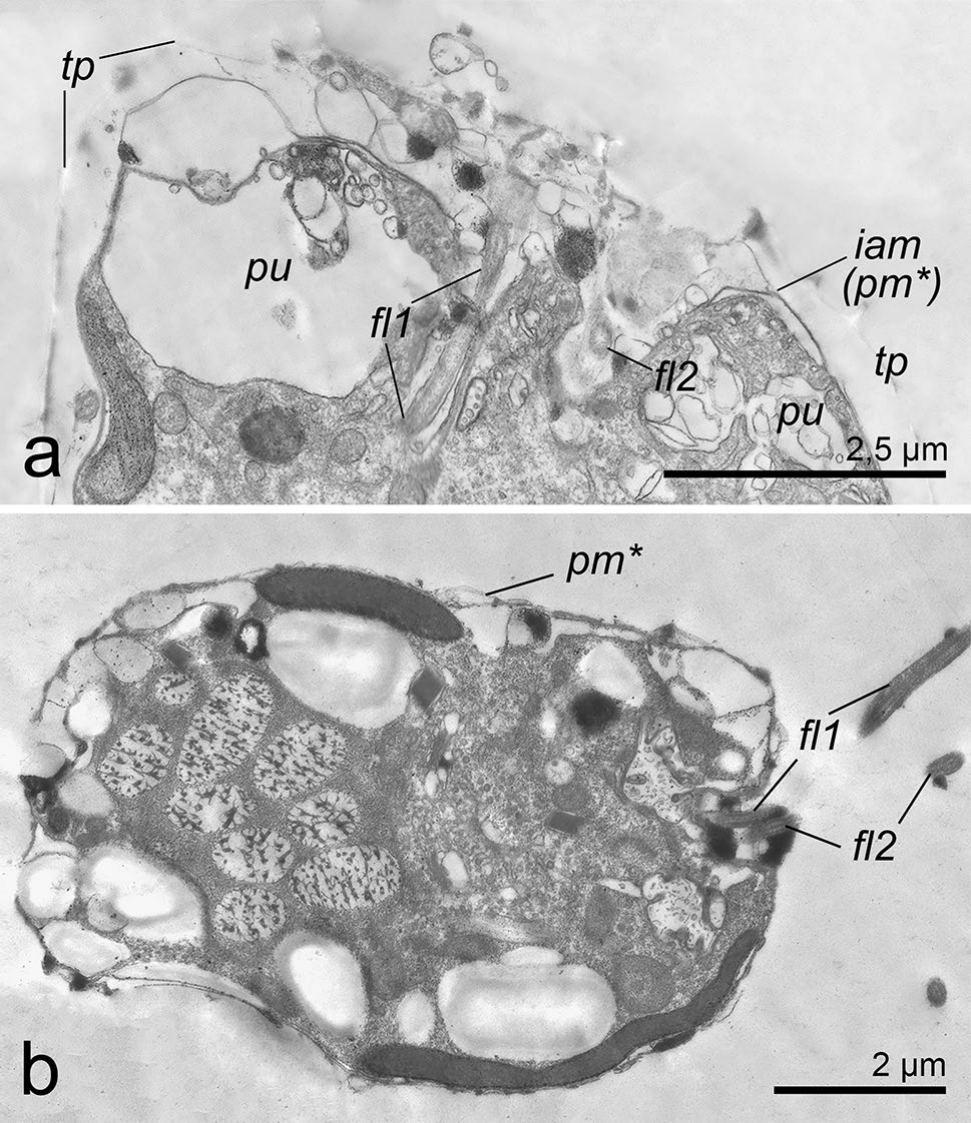
Flagella regeneration during ecdysis in cells of *P. cordatum*, strain RC CCMA 0466. (**a**): cell already has two new flagella in the moment of shedding of old thecal plates; (**b**): motile ecdysed cell with two flagella. fl1, fl2 – flagella, iam (pm*) – old inner amphiesmal vesicle membrane (new plasma membrane), pu – pusule, tp – thecal plates.

**Figure 11:**
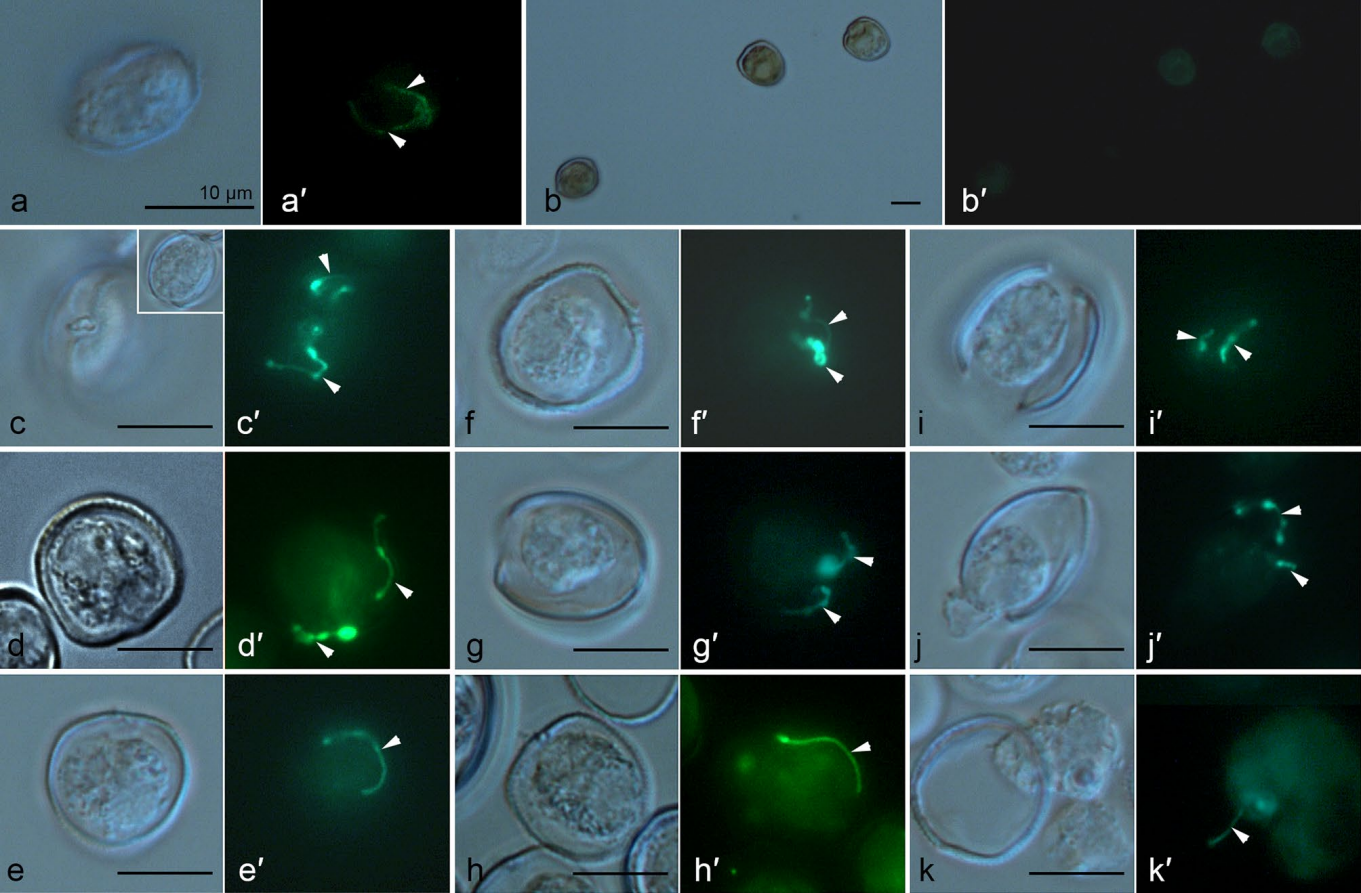
Cells of *P. cordatum*, strain CCAP 1136/16, at the different stages of ecdysis induced by centrifugation (10,000 g, 5 min) labelled by anti-α-tubulin antibodies to reveal flagella. (**a,a′**): untreated vegetative cell bearing flagella visualised by (**a**) differential interference contrast microscopy (DIC) and (**a′**) antibody labelling and fluorescent microscopy; (**b,b′**): cells which discarded flagella immediately after centrifugation visualised by (**b**) differential interference contrast microscopy (DIC) and (**b′**) antibody labelling and fluorescent microscopy; (**c–g′**): cells preparing to leave the old thecal plates in 4 h after centrifugation already possess flagella visualised by (**c–g**) differential interference contrast microscopy (DIC) and (**c′–g′**) antibody labelling and fluorescent microscopy, the inset on (**c**) shows an overview of a cell; (**h–k′**): cells leaving their old thecal plates in 4 h after centrifugation bear flagella visualised by (**h–k**) differential interference contrast microscopy (DIC) and (**h′–k′**) antibody labelling and fluorescent microscopy.

TEM images of cells treated by centrifugation demonstrated that at the moment of old theca shedding, new flagella were already formed (Fig. 10a), and a recently ecdysed cell had two functional flagella, albeit its amphiesma was not completely mature (Fig. 10b). This observation was confirmed by the anti-α-tubulin antibody labelling of *P. cordatum* cells at the different stages of the cell covering rearrangement. Control staining revealed flagella in intact vegetative cells (Fig. 11a,a′). Immediately after the induction of ecdysis by centrifugation, flagella were absent in cells that entered ecdysis (Fig. 11b,b′) but in 4 h cells preparing to leave the old thecal plates (Fig. 11c–h′), as well as cells escaping their old thecae had brightly stained flagella (Fig. 11i–k′).

## Discussion

### Not all external stressors induce ecdysis in *Prorocentrum cordatum*

We chose the stressor levels to induce ecdysis in a large fraction of *P. cordatum* cells and thus get a measurable response to identify the range of ecdysis triggers for this species and develop a good model system for the future research on this process. Some of the stressors, especially centrifugation at 10,000 g, were very strong. For this reason, the quantitative results of our experiments should not be directly extrapolated to the natural systems.

Nevertheless, most of the studied triggers to some extent mimic changes of conditions that can be encountered by dinoflagellates in the natural environment. Therefore, we can suggest that such stressors as mechanical agitation, changes in temperature, pH, and salinity also induce ecdysis in natural *P. cordatum* populations. Ecdysing cells similar to those described in this work were found in a natural bloom of *P. cordatum*^34^. The lower intensity of the natural triggers compared to the ones applied in this work would probably result in a smaller fraction of a population entering ecdysis, as it was shown experimentally for centrifugation. In our experiments, centrifugation at 500 g resulted in lower ecdysis relative to centrifugation at 5000 and 10,000 g, and vortexing for 1 min was not sufficient to induce ecdysis at all. Vortexing even suppressed the ecdysis rate relative to the control, which can be explained by a temporary loss of activity (such cells were immotile but still carried flagella) by dinoflagellates preventing progression of the ecdysis process. This is in agreement with an observation that already in 6 h after the treatment, the rate of ecdysis in the vortexed culture was not different from the control.

Centrifugation and vortexing represent examples of mechanical stressors which are known to affect dinoflagellate growth and proliferation both in culture and in nature. For example, dinoflagellates are sensitive to shaking and mixing, which hampers continuous culturing of these organisms^35–37^. It was suggested that some dinoflagellate species produce thin-walled cysts due to the excessive turbulence generated by wind and wave activity which are typical for the nearshore waters and regions of upwelling^38–41^. Although it is difficult to compare centrifugation/vortexing and turbulence directly, all these effects generate fluid shear stress which was shown to influence membrane fluidity in the dinoflagellate *Lingulodinium polyedra*^42^. The fact that in our experiments, centrifugation at 500 g induced ecdysis in a smaller fraction of a population compared to the higher centrifugation intensity can be explained by weaker fluid shear stress generated in this case. Nevertheless, *P. cordatum* cells successfully survived even centrifugation at 10,000 g, and the mortality rate in the centrifuged cultures was the lowest among all treatments applied. Thus, ecdysis is an effective strategy to resist this type of stressors.

Temperature decrease is also a trigger of encystment in dinoflagellates^23,24,26^. In natural systems, it occurs during transition from one season to another, abrupt weather fluctuations or as a result of cold water masses inflow. Similar to centrifugation cooling of a culture on ice turned out to be a very effective ecdysis trigger. In contrast, temperature elevation up to 35 ℃ did not result in ecdysis, probably because on a short time scale (20 min) this treatment does not represent a serious stressor to cells.

*P. cordatum* is known to live under salinities ranging from 2 to 35^31,43–45^. However, the abrupt shifts in conditions are usually more damaging than the gradual ones. Fast salinity changes periodically occur in natural habitats, for instance, in estuaries, due to wind mixing and tidal activity. In our experiments, both sharp increase and decrease in salinity induced ecdysis, but cells started to shed their thecae only in 4–6 h after the salinity change and compared to the other ecdysis triggers the response was rather week. Mortality rate in these treatments was at the same level as the rate of complete ecdysis; probably cells that had undergone ecdysis and lost their thecae providing rigidity to a cell covering could not survive changes in osmotic pressure accompanying salinity shifts.

In addition, we tested the effect of certain chemical compounds, tetracycline and DCB. Tetracycline and its derivatives represent bacteriostatic antibiotics widely used in aquaculture to suppress the spread of infectious diseases among cultured organisms^46,47^. Therefore, the surprising finding that tetracycline lowers the rate of ecdysis in untreated laboratory populations of *P. cordatum* while cells remain active and healthy may be relevant for the regions of aquaculture with a high concentration of tetracycline in water. If tetracycline reduces the ecdysis rate in *P. cordatum* cultures under normal culturing conditions, this compound may affect dinoflagellate dynamics in the regions of tetracyclines-treated aquaculture. The mechanism behind tetracycline-mediated suppression of ecdysis is not clear, but it was demonstrated that as a general rule tetracyclines impose various effects on the physiology of eukaryotic organisms, e.g. via regulation of gene expression by tetracycline-sensitive promoters and violation of mitochondrial translation^48–50^.

DCB is unlikely to be encountered by dinoflagellates in nature; however, being an effective inhibitor of cellulose synthesis^51^, it is used to study dinoflagellates in the laboratory with applications ranging from the investigations of their cell and life cycles to recording of their ion channels^32,52,53^. In combination with the ability to suppress cellulose synthesis the second activity of this substance – induction of ecdysis – makes DCB a potent tool to study cell covering rearrangement and its molecular mechanisms.

### Ecdysis and cysts of *P. cordatum*

**Figure 12:**
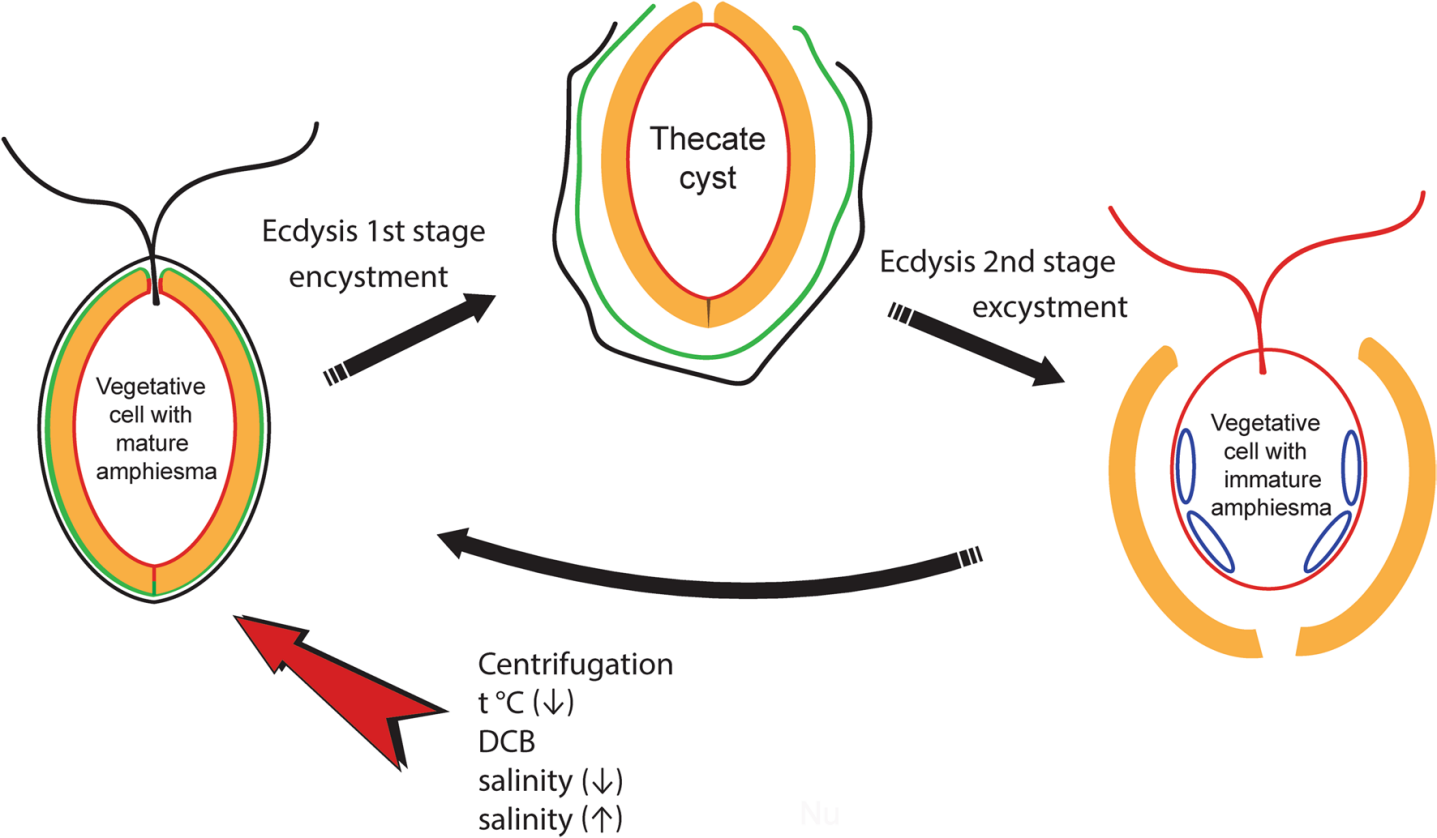
Schematic representation of the cell covering rearrangement accompanying encystment and excystment of *P. cordatum*. Black line – plasma membrane, green line – outer amphiesmal vesicle membranes, red line – inner amphiesmal vesicle membranes that become a new plasma membrane during ecdysis, blue line – juvenile amphiesmal vesicles. Thecal plates are depicted in orange colour.

Our data indicate that ecdysis in *P. cordatum* proceeds in two main stages separated by a relatively long period of time (Fig. 12). At the first stage, the plasma membrane, flagella and the outer amphiesmal vesicle membranes are shed. This happens immediately or very soon after the treatments implying a very fast mechanism behind simultaneous fusion of amphiesmal vesicles and discarding of the structures mentioned above. At the end of this stage, a cell is immotile, surrounded by cellulose thecal plates exposed to the environment and the new plasma membrane (former inner amphiesmal vesicle membranes) ensuring cell integrity and sustainability. At the second stage, which in our experiments usually began several hours after the completion of the first stage, thecal plates are shed leaving a naked cell that subsequently restores full amphiesma. These stages and ultrastructural changes accompanying them are the same independently of the *P. cordatum* strain used in the experiments. Moreover, they are very similar for the different ecdysis triggers applied. The omission of the cellulose accumulation and new thecal plates formation in the ecdysed cells induced by the DCB treatment is related to cellulose synthesis inhibition by this compound. Our observations indicate that the process of ecdysis may be under the control of a signaling pathway executed by signals coming from various types of receptors (e.g. mechanoreceptors, temperature receptors, chemical receptors). According to this scenario, the plasma membrane integrity is compromised only after the activation of a signaling pathway involved in ecdysis control. Alternatively, ecdysis may represent an unspecific response to the membrane damage caused by applied stressors directly and independently of the stressor nature. In this case, violation of the plasma membrane integrity is a signal triggering the rest stages of the cell covering rearrangement during ecdysis. A combination of both scenarios cannot be excluded.

Often thin-walled cysts of dinoflagellates are produced as a result of the complete ecdysis^10,14,20,54^. During the cyst formation, a cell leaves its old theca and then persists as an immotile stage surrounded by a special protecting layer termed the pellicle until excystment. Based on these facts, thin-walled cysts are also called “ecdysal” and “pellicle”. We argue that in our experiments *P. cordatum* cysts were the cells that entered ecdysis by discarding their plasma membrane, flagella and the outer amphiesmal vesicles membranes, while theca shedding represented excystment rather than encystment (Fig. 12). Indeed, TEM images of a cell leaving its old theca and a cell with a recently shed theca (ecdysed cell), as well as antibody labelling of flagella in cells preparing to leave the old thecal plates and cells discarding their thecae explicitly demonstrated that such cells already possessed new flagella. The only period when dinoflagellates were immotile and seemingly inactive was the period following the initiation of ecdysis by membrane shedding, which was confirmed by the intravital observations. Therefore, in line with the definition of cysts as non-flagellated benthic stages of dinoflagellates^20^, we consider cells at this ecdysis stage as cysts. Since by the time of theca shedding, *P. cordatum* cells restored flagella and motility, they should not be considered as cysts according to the traditional definition mentioned above.

However, data on diverse life stages of dinoflagellates obtained over the last years, including such peculiar findings as the division of non-motile stages (division cysts) and flagellar motility in presumable cysts, have revived the discussion of the cyst concept^55^. Recently Salgado et al.^55^ described benthic stages of the dinoflagellates *Protocerarium reticulatum* and *Ceratocorys mariaovidiorum* which displayed characteristic features of cysts (e.g. motionless behaviour, reduction of cytoplasm and condensation of chloroplasts) but possessed flagella and were able to move when disturbed. In light of this discovery, there is a possibility that flagellated *P. cordatum* cells released from their old thecae could also be a special type of cysts. Nevertheless, we believe that they represented vegetative cells, because unlike benthic stages described by Salgado et al.^55^, ecdysed *P. minimum* cells did not show morphological traits typical to dinoflagellate cysts, looked very similar to vegetative cells, and had general amphiesma structure characteristic to the latter. Moreover, the appearance of biflagellate cells as a result of theca shedding was noticed in the natural bloom of *P. cordatum* in the Gulf of California^39^.

Interestingly, Bravo et al.^14^ reported thecate cysts of *Alexandrium minutum* corresponding to ecdysal cysts of the same species and those of *Alexandrium tamutum* described in Figueroa et al.^56^. Such thecate cysts seem to be very similar to *P. cordatum* cysts observed in this work in terms of morphology and excystment via theca shedding. Therefore, we think that the term “thecate cyst” suggested by Bravo et al.^14^ can be applied to the *P. cordatum* cysts described here.

The trait that distinguishes thecate cysts of *P. cordatum* from thin-walled (pellicle) cysts of other dinoflagellate species is the absence of the non-membrane pellicular layer. The pellicular layer is a distinct fibrous layer present in the cell covering of thin-walled cysts and sometimes vegetative cells of various dinoflagellates^7,9–12^. In vegetative cells, it is located inside amphiesmal vesicles, whereas in pellicle cysts, it is found outward from the new plasma membrane originating from the inner amphiesmal vesicle membranes (sometimes termed “pellicle membrane”). The chemical nature and structure of the pellicular layer are not clear yet. It was suggested that this layer contained sporopollenin-like, cellulosic and protein components^4,5,10,12^. It is generally assumed that the pellicular layer provides rigidity and resistance to a cyst^20^. In our work, the pellicular layer in the cell covering of vegetative cells and putative thecate cysts of *P. cordatum* was not identified by TEM. Indeed, no layer similar to the pellicular layers of different dinoflagellates species described in the literature^4,5,7,10–12^ was observed in cells.

It is worth noting that variable terminology regarding thin-walled cysts of dinoflagllates has generated some confusion over the last decades. There is still no consensus on the best term to refer to these stages which were called “temporary”, “ecdysal” and “pellicle” by different researchers based on their relatively short duration, ecdysal origin and pellicle-containing wall, respectively (reviewed in^20^). The picture is further complicated by the existence of thecate cysts which possibly represent a different type of quiescent life stage of dinoflagellates. Indeed, thecate cysts of *A. minutum* were found in addition to the thick-walled resting and thin-walled pellicle cysts of the same species^14^. At the present time, it is not quite clear whether *P. cordatum* is able to produce resting and pellicle cyst along with the thecate ones. There are only a few previously published research works reporting *P. cordatum* cysts. Grzebyk and Berland^25^ used scanning electron microscopy to image temporary cysts produced as a result of the prolonged temperature decrease in the culture of this species and showed a layer surrounding a cyst that they considered a pellicular envelope. Manoharan et al.^57^ mention immotile cells with thickened cell walls in the *P. cordatum* cultures grown in the dark for more than eight days. Besides, Martínez-López et al.^34^ found cysts in the natural bloom of *P. cordatum*. They referred to such cysts as temporary but we attribute them to thecate cysts based on the provided light microscopy pictures. It is probable that prolonged impacts cause a different cyst type formation compared to the short-term stressors. A rigorous study of the different *P. cordatum* cyst types is a prospect for future research.

### *P. cordatum* as a model organism to study ecdysis

To date, molecular mechanisms of ecdysis remain unclear^19,20^. Meanwhile, this physiological process unique to dinoflagellates undoubtedly represents an effective adaptive strategy and thus has to be carefully investigated. In addition to its role in the transition from vegetative cells to cysts and vice versa, ecdysis may have other, so far unrecognised functions. The complex cell covering may impede conventional membrane renovation and other physiological processes, e.g. nutrition, in dinoflagellates^58^. Massive cell covering rearrangement during ecdysis may represent the way to adjust the molecular composition of the plasma membrane in accordance with current environmental conditions^13,19^. This hypothesis can be verified or disproved exclusively by the thorough research using techniques of cell biology.

We suggest *P. cordatum* as a model armoured dinoflagellate that can be effectively used to study cellular and molecular aspects of ecdysis. The present work provides several arguments for this suggestion. First, we showed that ecdysis in this species can be easily triggered by various short-term treatments. Even 5–20 min interventions resulted in a high rate of ecdysis, e.g. in the case of centrifugation, temperature decrease and application of DCB over 90% of cells started to ecdyse and over 40%—completed ecdysis in 6 h after the treatment. Notably, based on the centrifugation experiments, we conclude that the high ecdysis rate can be achieved independently of the culture growth phase. The ability to induce a high fraction of cells to ecdyse on demand is a prerequisite for various cell biology approaches requiring substantial biomass of target cells. Furthermore, our TEM data prove that cells respond to various triggers by the same changes in the arrangement of membranes within amphiesma and that such changes are not strain-specific. In addition, TEM images confirm that DCB not only induces ecdysis but also inhibits the formation of new thecal plates, which can be used in experiments to clarify the role of cellulose and thecal plates in ecdysis of armoured species. Finally, the lack of the pellicle layer in the thecate cysts makes *P. cordatum* a relatively simple model and allows focusing on the membrane rearrangement representing the essence of the ecdysis process. Taken together, these aspects should facilitate the investigation of cellular mechanisms of dinoflagellate ecdysis.

## Materials and methods

### Culture material and growth conditions

Cultures of *Prorocentrum cordatum*, strains CCAP 1136/16 and RC CCMA 0466, were maintained in artificial seawater-based f/2 medium^59^ containing no silicate and sterilised by autoclaving (vitamin mixture was sterilised by sterile filtration and added separately) at salinities of 25 and 17, respectively (salinity is reported using the Practical Salinity Scale approved by the Joint Panel of Oceanographic Tables and Standards, according to which salinity is defined as a pure ratio, and has no dimensions or units). The batch cultures were grown under a 12 h/12 h light/dark cycle at 100 μmol photons m^−2^ s^−1^ and 23–24 ˚C.

### Experimental procedures

For the conformity between all treatments and biological replicates we pre-incubated dinoflagellate cultures prior to the experimental manipulations. *P. cordatum* cells were inoculated into 50 ml glass flasks at a cell concentration of 40 × 10^3^ cells ml^-1^ and then were allowed to grow for 4 days to reach the exponential growth phase. During this time cell counts were performed daily. Experimental treatments applied on the fourth day included different types of disturbances: centrifugation for 5 min at 500 g, 5000 g, and 10000 g; vortexing for 1 min at the maximal power (V-1 plus, BioSan); temperature increase up to 35 ˚C for 20 min; temperature decrease – 20 min on ice; salinity increase up to 40; salinity decrease to 10; 0.5 pH increase; 0.5 pH decrease; addition of 150 µM 2,6-dichlorobenzonitrile (DCB) (Sigma-Aldrich, St. Louis, MO, USA) dissolved in dimethyl sulfoxide (DMSO) (Sigma-Aldrich, St. Louis, MO, USA); addition of DMSO; addition of tetracycline (Sigma-Aldrich, St. Louis, MO, USA) at 10 mg l^-1^. Shifts in salinity were ensured by the addition of either a concentrated sodium chloride solution or distilled water, shifts in pH – by the addition of sodium hydroxide or hydrochloric acid solutions. Importantly, the addition of all solutions was performed gradually, in 1–2 µl droplets with gentle mixing to avoid sharp local shifts in conditions. Vortexing for 1 min was included, because we used it to cease dinoflagellate motility temporally for the purposes of the intravital photography and cell counts and had to be sure that such treatment did not induce ecdysis itself.

Each treated culture sample was divided into several subsamples to estimate the rate of ecdysis achieved in 2, 4, and 6 h after the treatment, the fraction of viable cells immediately after and after 4 h following the treatment and the fraction of cells that had entered ecdysis by discarding the plasma membrane.

### Analytical procedures

Cell counts were carried out by means of a Fuchs-Rosenthal counting chamber and a light microscope. The same approach was used for counting cells and empty thecae after incubation for 2, 4, and 6 h to estimate the rate of complete ecdysis as the fraction of cells that discarded their thecae according to the formula:

$$E_{thecae} = { }\frac{{N_{thecae} /2}}{{N_{cells} }}{ } \times 100{\text{\% ,}}$$

where *N*_*thecae*_ – number of empty thecae pieces, *N*_*cells*_ – number of cells. Essentially all empty thecae were separated due to vortexing applied to mix samples prior the filling of a counting chamber and thus appeared as halves during the counts; therefore, *N*_*thecae*_ was divided by a factor of 2 to obtain the number of discarded thecae.

The counts were performed using the phase contrast mode of a microscope to better visualise discarded thecae and in at least three technical replicates; at least 200 cells per sample were counted. It must be noted that for the estimation of the rate of ecdysis (*E*_*thecae*_) it is better to incubate samples in the glass vials, not in the plastic ones. Different types of plastic are characterised by different adhesive properties towards dinoflagellate cellulosic thecae, which can lead to the underestimation of thecae numbers and thus underestimation of the rate of ecdysis.

It must be noted that thecae shedding is the final stage of ecdysis preceded by shedding of the plasma and outer amphiesmal vesicle membranes and thus reflects only the completion of this process but cannot be used to estimate the rate of ecdysis initiation. To determine the fraction of cells entering ecdysis immediately after the treatment (discarding or partially discarding old plasma and outer amphiesmal vesicle membranes before the start of thecae shedding), one subsample was stained by the cellulose-specific fluorescent stain Calcofluor White M2R (Sigma-Aldrich, St. Louis, MO, USA). Then the stained cells were observed and photographed with a microscope Leica DM2500 (Leica-Microsystems, Germany) using phase contrast optics and under UV light. Cellulosic thecal plates of dinoflagellates are not stained if a plasma membrane is intact, whereas cells that have entered ecdysis by discarding or partially discarding their old plasma membranes are characterised by the well-stained thecal plates^60^. Thus, the fraction of cells encircled by fluorescently stained thecal plates reflected the fraction of cells that had entered ecdysis by discarding the plasma membrane immediately after or during the treatment applied. Ecdysis rate determined by Calcofluor White M2R staining before the completion of ecdysis (thecae shedding) is further designated as *E*_*calcofluor*_.

Cell mortality was determined immediately and in 4 h after the treatments by propidium iodide (PI) (Sigma-Aldrich, St. Louis, MO, USA) staining followed by observation and photography using a fluorescent microscope Leica DM2500 (Leica-Microsystems, Germany) or Zeiss Pascal Axiovert 200 M (Carl Zeiss, Germany). The approach is based on the fact that PI stains nuclear DNA of dying and dead cells, since it can pass through the plasma membrane only if its integrity was irreversibly disrupted^61, 62^. Thus, the fraction of cells stained by propidium iodide represents the fraction of dead cells. The digital images were analysed, cropped and measured using ImageJ (https://imagej.nih.gov/ij/). The Adobe Photoshop 21.0.1 (https://www.adobe.com/) software was used for images labelling and arranging into the plates. In addition, after the experimental treatment the number of cells in some subsamples was counted. These subsamples were incubated for 24 h and then motility of cells was inspected microscopically and cell counts were performed again in order to find out whether some cells were lysed.

### Transmission electron microscopy (TEM)

For the electron microscopy studies, we used f/2 medium with salinity 17 or 25 for fixatives dissolution and rinsing of samples.

To study the cell covering rearrangement after centrifugation (10,000 g for 5 min), cells of *P. cordatum*, CCAP 1136/16 and RC CCMA 0466 strains, were fixed immediately after centrifugation and 4 h after the treatment. The 4 h cells were harvested by centrifugation (1200 g for 3 min) prior to fixation. The cells were fixed in 2,5% (v/v) glutaraldehyde (Sigma-Aldrich, USA) at 4 °C for 40 min, then rinsed and postfixed in 1% (w/v) osmium tetraoxide (TAAB Laboratories Equipment, UK) at room temperature for 1 h. After rinsing the pellets were embedded in 2% agar, dehydrated through a graded series of ethanol, infiltrated with acetone-resin mixtures and embedded in Epon 812 – Araldite M (Fluka, Switzerland) resin mixture. Ultrathin sections were cut using an Ultracut E (Reichert Jung, Austria) ultramicrotome, contrasted with uranyl acetate and lead citrate and examined in a Libra 120 (Carl Zeiss, Germany) microscope. To study the effect of DCB treatment on *P. cordatum* cells, we also fixed them immediately after the addition of the agent into the sample and in 4 h. The cell suspensions were incubated with glutaraldehyde, which was added directly to the culture medium (final concentration 2.5%), at 4 °C for 40 min. Then f/2 medium was added and the cells were harvested by centrifugation (1200 g for 3 min). After rinsing with fresh portions of f/2 medium, samples were postfixed in 1% (w/v) osmium tetraoxide at room temperature for 1 h and treated according to the procedure described above.

Non-treated cells were fixed and served as the control. The glutaraldehyde stock solution was added to the sample to reach a final concentration of 2.5%; fixation and subsequent treatment were carried out in the same way.

The electron micrographs were cropped, adjusted, labelled and arranged into the plates with Adobe Photoshop 21.0.1 (https://www.adobe.com/). The change overall image contrast and brightness, sharpening filter and “Dodge” tool (lightening) were applied.

### Antibody labelling

To determine the presence of flagella in the dinoflagellate cells at the different stages of cell covering rearrangement, the cells were fixed immediately after centrifugation and 4 h after the treatment. The non-treated cells were also fixed as a control. The cell suspensions were incubated with formaldehyde solution added directly to the culture medium (final concentration 4%) for 5 min, then harvested gently by centrifugation at 600 g for 1 min and fixed in 4% formaldehyde in 0.1 M phosphate buffered saline (PBS) at 4 °C for 30 min. After fixation, the cells were washed in 0.1 M PBS containing 100 mM glycine at room temperature for 10 min. Then they were treated by a mixture of 1% bovine serum albumin (BSA) (A-0847, AppliChem, Darmstadt, Germany) and 0.1% Triton X-100 (Sigma-Aldrich, St. Louis, MO, USA) in 0.1 M PBS at room temperature for 10 min to prevent non-specific antibody binding and ensure membrane permeabilization. Then samples were rinsed in 0.1 M PBS thrice. The primary monoclonal mouse anti-α-tubulin antibodies (T5168, Sigma-Aldrich, St. Louis, MO, USA) tested in a previous work^63^ diluted in 0.1 M PBS/1% BSA (1:100) were used for immunolabelling. The incubation was carried out at 4 °C overnight. After rinsing in PBS, the cells were incubated with secondary goat anti-mouse antibodies conjugated with fluorescein isothiocyanate (FITC) (F9006, Sigma-Aldrich, St. Louis, MO, USA) diluted to 1:80 in 0.1 M PBS at room temperature for 2 h. After rinsing in PBS, preparations were embedded in the glycerol-PBS mixture and examined by the light microscope Leica DM2500 (Leica-Microsystems, Germany) using differential interference contrast and fluorescence (UV light and L5 filter cube). Obtained images were cropped using ImageJ (https://imagej.nih.gov/ij/). The Adobe Photoshop 21.0.1 (https://www.adobe.com/) software was used to adjust relative contrast and brightness of images.

### Video acquisition

Live observations of the ecdysis process were video recorded using a Micromed I inverted microscope (Micromed, Saint-Petersburg, Russian Federation) equipped with a ToupCam 9.0 MP digital camera and ToupView 3.7 software (Hangzhou ToupTek Photonics Co., Ltd, Zhejiang, P.R. China, https://touptek.com/). The video with dimensions of 3488 × 2616 px and rate of 2 frames per second were initially acquired. The video fragments of interest were extracted, cropped and speeded up till 25 or 30 frames per second using Movavi Editor Plus 2020 software (https://www.movavi.ru/).

### Statistical analysis

MaxStat Lite 3.60 software (https://maxstat.de/en/home-en/) was used for the statistical analysis. Mean values were compared using the unpaired two-tailed t-test or ANOVA with Tukey post-test.

## Supplementary information

Supplementary Information 1.

Supplementary Video 1.

Supplementary Video 2.

## Data Availability

The datasets generated and analysed during this study are available in the Zenodo repository, https://doi.org/10.5281/zenodo.4011649.
