# Supplementary material for: Stressor-induced ecdysis and thecate cyst formation in the armoured dinoflagellates Prorocentrum cordatum
Source: Sci Rep. 2020 Oct 27;10:18322. doi: 10.1038/s41598-020-75194-3 (PMC7591879; doi:10.1038/s41598-020-75194-3)
Supplement: Supplementary file 1 — Supplementary Information 1. [file 41598_2020_75194_MOESM1_ESM.docx]

Supplementary Materials

**Stressor-induced ecdysis and thecate cyst formation in the armoured dinoflagellates *Prorocentrum cordatum***

Olga Matantseva^*^, Mariia Berdieva, Vera Kalinina, Ilya Pozdnyakov, Sofia Pechkovskaya and Sergei Skarlato

Laboratory of cytology of unicellular organisms, Institute of Cytology of the Russian Academy of Sciences, Saint Petersburg, Russia

^*^ Correspondence: [matantseva@incras.ru](mailto:matantseva@incras.ru)

**Supplementary Materials legends**

**Supplementary Video 1.** Ecdysing cell of *Prorocentrum cordatum.* The process of theca shedding is shown.

**Supplementary Video 2.** Ecdysed cell of *Prorocentrum cordatum.* Following theca shedding, the cell is motile, although moves slowly because of the stress by heating during microscopic observations.
